# A Survey of Chloroplast Protein Kinases and Phosphatases in *Arabidopsis thaliana*

**DOI:** 10.2174/138920208784340740

**Published:** 2008-05

**Authors:** I Schliebner, M Pribil, J Zühlke, A Dietzmann, D Leister

**Affiliations:** Lehrstuhl für Botanik, Department Biologie, Ludwig-Maximilians-Universität München, Menzinger Str. 67, 80638 München, Germany

**Keywords:** *Arabidopsis thaliana*, chloroplast, chloroplast transit peptide, protein kinase, protein phosphatase, protein phosphorylation, proteomics.

## Abstract

Protein phosphorylation is a major mode of regulation of metabolism, gene expression and cell architecture. In chloroplasts, reversible phosphorylation of proteins is known to regulate a number of prominent processes, for instance photosynthesis, gene expression and starch metabolism. The complements of the involved chloroplast protein kinases (cpPKs) and phosphatases (cpPPs) are largely unknown, except 6 proteins (4 cpPKs and 2 cpPPs) which have been experimentally identified so far. We employed combinations of programs predicting N-terminal chloroplast transit peptides (cTPs) to identify 45 tentative cpPKs and 21 tentative cpPPs. However, test sets of 9 tentative cpPKs and 13 tentative cpPPs contain only 2 and 7 genuine cpPKs and cpPPs, respectively, based on experimental subcellular localization of their N-termini fused to the reporter protein RFP. Taken together, the set of enzymes known to be involved in the reversible phosphorylation of chloroplast proteins in *A. thaliana* comprises altogether now 6 cpPKs and 9 cpPPs, the function of which needs to be determined in future by functional genomics approaches. This includes the calcium-regulated PK CIPK13 which we found to be located in the chloroplast, indicating that calcium-dependent signal transduction pathways also operate in this organelle.

## INTRODUCTION

Phosphorylation of amino acid side chains can modulate the conformation, activity, localization and stability of proteins, and around one-third of all eukaryotic proteins are thought to be reversibly phosphorylated [1]. Therefore, protein phosphorylation plays a central role in regulating cellular functions, particularly in signal transduction pathways (reviewed in: [2, 3]). The reversible phosphorylation of proteins involves two types of enzymes: protein kinases (PKs) and phosphatases (PPs). In signaling pathways, PKs can be arranged in cascades, allowing amplification, feedback, cross-talk and branching in the transduction of the signal [2, 4, 5].

Various types of PKs and PPs exist in plants, classified according to the presence of domains in addition to the catalytic domain mediating (de)phosphorylation (e.g. receptor kinases), their regulation (e.g. Ca^2+^/calmodulin-dependent kinases) or their amino acid substrate (e.g. serine/threonine, tyrosine or histidine kinases) [4, 6-15]. The complete genome sequence of the model plant *Arabidopsis thaliana* allowed to survey comprehensively its total complement of PKs and PPs, resulting in more than 800 PKs [16] and 112 PPs [9] so far. As of early 2008, in the PlantsP [17] and TAIR [18, 19] databases, 970 PKs and 217 PPs were listed for *A. thaliana*.

The chloroplast, the characteristic organelle of green algae and plants, is thought to contain around 3000 different proteins in the model plant species *A. thaliana* [20, 21]. Among those, a number is reversibly phosphorylated including proteins involved in the photosynthetic light reaction [22, 23], starch metabolism [24] and transcription [25, 26]. However, only few chloroplast PKs (cpPKs) and PPs (cpPPs) have been experimentally identified in *A. thaliana* and other flowering plants so far. Here we will summarize current knowledge on *Arabidopsis* cpPKs and cpPPs and present genomic approaches for the systematic identification and characterization of the chloroplast complement of PKs and PPs.

## EXPERIMENTALLY CHARACTERIZED cpPKs AND cpPPs

The reversible phosphorylation of thylakoid proteins has been associated with the regulation of the migration between the photosystems of LHCII, the light-harvesting complex of photosystem II (PSII) [27, 28], as well as with the turnover of PSII proteins [29, 30]. Based on sequence homology to the cpPK Stt7, isolated from the green alga *Chlamydomonas reinhardtii*, the two *A. thaliana* cpPKs STN7 and STN8 have been identified [31] and their thylakoid localization has been experimentally shown [32, 33] (Table **1**). The analysis of loss-of-function mutants showed that STN7 is required for LHCII phosphorylation and state transitions [32, 33], whereas STN8 is necessary for the reversible phosphorylation of the PSII proteins D1, D2, CP43, PSII-H and CP29 [33, 34]. Further biochemical analyses will be required to clarify whether STN7 and STN8 directly phosphorylate photosynthetic proteins, or whether they operate in phosphorylation cascades involving additional thylakoid PKs. Additional members of such phosphorylation cascades might be represented by the so-called thylakoid-associated PKs TAK1, 2 and 3 [35, 36] (Table **1**). However, in contrast to STN7 and STN8 these proteins lack a chloroplast transit peptide (cTP). 

The chloroplast α subunit of casein kinase 2, cpCK2α, has been originally identified in mustard (*Sinapis alba* L.). cpCK2α phosphorylates *in vitro* components of the plastid transcription apparatus [37] and the corresponding orthologue has been also detected in chloroplasts of *A. thaliana* [38] (Table **1**). Three further cpPKs have been tentatively identified during two proteomic studies [39, 40] but their localization has not been confirmed by independent approaches yet (Table **1**). 

Only two cpPPs have been identified so far. The dual-specificity protein phosphatase DSP/SEX4 can bind to starch granules and is involved in the regulation of starch metabolism [41-43]. AtRP1 exhibits bifunctional PK/PP properties and is capable of (de)phosphorylation of the regulatory threonine residue of Arabidopsis pyruvate, orthophosphate dikinase (PPDK) [44]. 

## GENOME-WIDE PREDICTION OF cpPKs AND cpPPs

The vast majority of chloroplast proteins are targeted to the organelle by their N-terminal signal sequences, the chloroplast transit peptide (cTP), and imported via the Tic/Toc translocon [45]. For the prediction of cTPs various algorithms have been developed, and the accuracy of prediction can be further improved by combining several predictors. Thus, the specificity of combinatorial cTP prediction increases with the number of predictors used; as expected, this gain in specificity occurs at the expense of sensitivity [21]. We have employed nine different algorithms for cTP prediction and all the four cTP-containing cpPKs and cpPPs, which were unambiguously identified experimentally, namely STN7, STN8, cpCK2α, AtRP1 and DSP4/SEX4, were correctly predicted by at least 6 of the 9 predictors (Table **1**). Applying the “6 of the 9” predictor combination to the entire complement of 970 PKs and 217 PPs encoded in the nuclear genome of A. thaliana, 45 PKs and 21 PPs should contain a cTP (Table **2**). Remarkably, among the cpPKs predicted by the 6/9 approach, transmembrane (TM)-receptor PKs and related PKs are relatively under-represented, whereas non-TM PKs are clearly over-represented (Table **2**). Among the predicted cpPPs, serine/threonine phosphatases are under-represented but PP 2C-type PPs predominate (Table **2**).

## EXPERIMENTAL VALIDATION OF A REPRESENTATIVE SET OF PREDICTED cpPKs AND cpPPs

**Fig. (1):**
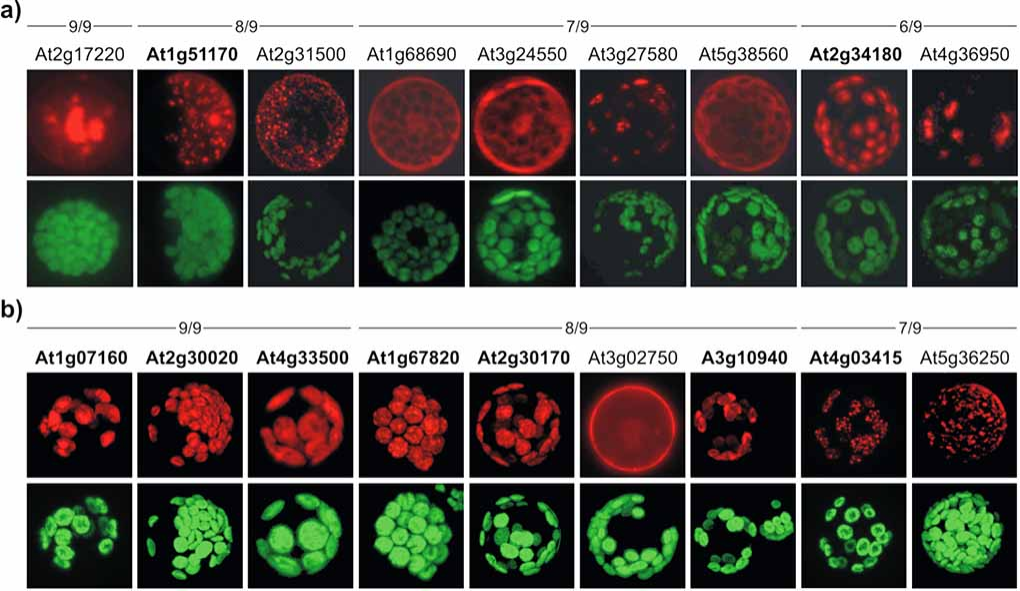
Fluorescence micrographs of *A. thaliana* protoplasts transfected with N-terminal fusions of the predicted transit peptides of the respective cpPKs (**a**) and cpPPs (**b**) to dsRED. The pictures are presented in false color with RFP fluorescence shown in red and chlorophyll autofluorescence in green. The predictor combinations are provided above each panel and chloroplast-located proteins are indicated in bold.

How many of the tentative cpPKs and cpPPs found by the 6/9 approach are truly located in the chloroplast? To answer this question, the N-terminal regions of the proteins containing the tentative cTPs were fused to the red-fluorescent protein (RFP) [46] and transfected into Arabidopsis protoplasts. Comparison of the position of signals from the RFP fusions and from chlorophyll autofluorescence allowed to validate the chloroplast location of the tentative cpPPs and cpPKs (Fig. **1**). Of the 9 predicted cpPKs tested, only two proteins actually exhibited a chloroplast location, notably At1g51170 and At2g34180 both belonging to class 4 of non-TM PKs (Table **3**). Interestingly, At2g34180 is annotated as “CBL (calcineurin B-like calcium sensor) protein interacting protein kinase 13” (CIPK13) and contains a NAF domain thought to mediate the interaction with CBL proteins (Table **4**). This supports the view that calcium-dependent signal transduction pathways also operate in chloroplasts. This is in line with the recent finding of calcium regulation of chloroplast protein translocation [47, 48]. 

In contrast to cpPKs, a large fraction of tentative cpPPs are truly located in the chloroplast; thus, for 7 of the 13 tentative cpPPs a chloroplast location could be confirmed (Table **3**). Taken together, identification of cpPKs by cTP prediction is very error-prone, whereas cpPPs could be identified in a relatively reliable way. One can only speculate why the cTP prediction produced so many false positive results for PKs. A possible explanation is that the kinase domain of the proteins interferes with cTP prediction; at least in one case (At4g36950) the falsely predicted cTP overlapped with the kinase domain. 

## CONCLUDING REMARKS

Around 10% of the nuclear genes in Arabidopsis are estimated to encode chloroplast proteins [20]. If this extrapolation is extended to major protein families such as protein kinases and phosphatases, more than 100 cpPKs and 20 cpPPs should operate in the photosynthetic organelle. Although our genomic approach more than doubled the set of known enzymes involved in reversible chloroplast protein phosphorylation, the current number of 6 confirmed cpPKs and 9 cpPPs is still much smaller than the one based on the genome-wide extrapolation described above and also much smaller than expected based on the cTP predictions of PKs and PPs (see Table **2**).

Although the chloroplast -- as an endosymbiotic organelle derived from a prokaryotic ancestor -- provided the eukaryotic cell with the two-component receptor family (which is related to the bacterial two-component histidine kinase receptors), it is tempting to speculate that the photosynthetic organelle might have failed to quantitatively adopt in return the eukaryotic concept of reversible protein phosphorylation at serine and threonine residues. This could mean that the actual number of PKs and PPs in the chloroplast is markedly lower than expected. Nevertheless, only approaches combining bioinformatic prediction and experimental validation of subcellular location of proteins as outlined in this work will systematically contribute to identify the entire complement of chloroplast protein kinases and phosphatases.

## Figures and Tables

**Table 1:** Experimentally Identified cpPKs and cpPPs in *A. thaliana*

Accession Number	Designation	Group a	Prediction Ratio	Experimental Approach	Reference
cpPKs					
At1g68830	STN7	none b	9/9	RFP/GFP fusion, *in-vitro* import, immunolocalization	[32, 33]
At5g01920	STN8	none b	8/9	RFP fusion,	[33, 34]
At4g02630	TAK1	1.6.3	1/9	Immunolocalisation,*in-vitro* import c	[35, 36]
At1g01540	TAK2	1.6.3	4/9	Sequence homology based d	
At4g01330	TAK3	1.6.3	3/9		
At2g23070	cpCK2α	4.5.3	6/9	GFP fusion	[38]
At3g44610	PK-like protein	4.2.6	5/9	proteomics	[40]
At5g20930	TSL (TOUSLED)	none b	4/9		
At1g76370	Putative PK	1.2.2	3/9		[39]
**cpPPs**					
At3g52180	DSP4/SEX4	6.2	7/9	GFP fusion, immunolocalization	[41-43]
At4g21210	AtRP1	6.5	9/9	GFP fusion	[44]

Predictions of chloroplast targeting were performed with the following algorithms: Predotar [49], TargetP [50], Protein Prowler [51], AAIndexLOC [52], PredSL [53], SLP-Local [54], WoLF PSORT [55], MultiLOC [56] and PCLR [57]. Several combinations were tried, in which a PK was considered to be chloroplast located when predicted by at least *n* (‘*n* of 9’) of the nine predictors, with *n* ranging from 1 to 9.

a according to PlantsP Kinase and Phosphatase database (http://plantsp.genomics.purdue.edu/html/families.html);

b not classified yet;

c TAK1 does not posses a cleavable cTP;

d chloroplast location was not reproducible (Bonardi, Pesaresi, Becker, Schleiff, Leister, unpublished data).

**Table 2:** Number of Predicted cpPKs and cpPPs in *A. thaliana*

Group	Definition	Entire Genome	Predicted cpPKs/cpPPs								
			1/9	2/9	3/9	4/9	5/9	6/9	7/9	8/9	9/9
**PKs**											
1	Transmembrane (TM) receptor and related non-TM	558	263	104	58	35	26	15	12	6	4
2	ATN1/CTR1/EDR1/GmPK6- like	52	28	12	5	4	4	1	0	0	0
3	Casein kinase I	16	5	2	1	0	0	0	0	0	0
4	Non-TM	278	159	82	62	45	36	23	11	4	1
5	other	4	2	0	0	0	0	0	0	0	0
-	not classified yet	62	34	22	15	11	6	6	5	5	2
total		970	491	222	141	95	72	45	28	15	7
**PPs**											
6.1	Ser/Thr	94	38	16	8	5	3	1	1	1	1
6.2	Dual-specific	23	14	8	6	6	6	5	3	1	0
6.3	PP 2C-type	89	64	38	27	19	15	12	12	9	5
6.4	Tyr	6	3	3	3	3	2	2	2	1	0
6.5	other	5	3	3	2	2	2	1	1	1	1
total		217	122	68	46	35	28	21	19	13	7

PK/PP identifiers and classifications were retrieved from the PlantsP database (http://plantsp.genomics.purdue.edu) and protein sequences from TAIR (http://www.arabidopsis.org/; genome release 7). Prediction of cTPs was performed as described in Table **1**. Ser, serine; Thr, threonine; Tyr, tyrosine.

**Table 3:** *In-Vivo* Localisation of RFP-Fusion Proteins in *A. thaliana* Protoplasts

Accession Number	Class	Prediction Ratio	Localization of RFP-Fusion
cpPKs			
At2g17220	1	9/9	elsewhere
At1g51170	4	8/9	cp
At2g31500	4	8/9	probably mt
At1g68690	1	7/9	cyt
At3g24550	1	7/9	cyt a
At3g27580	4	7/9	elsewhere
At5g38560	1	7/9	cyt a
At2g34180	4	6/9	cp
At4g36950	4	6/9	elsewhere
**cpPPs**			
At1g07160	6.3	9/9	cp
At2g30020	6.3	9/9	cp
At4g33500	6.3	9/9	cp
At1g67820	6.3	8/9	cp
At2g30170	6.3	8/9	cp
At3g02750	6.3	8/9	cyt
At3g10940	6.2	8/9	cp
At5g66720	6.3	8/9	probably cyt + mt
At1g71860	6.4	7/9	n [58]
At2g35350	6.3	7/9	cyt
At4g03415	6.3	7/9	cp
At5g36250	6.3	7/9	mt
At3g23610	6.2	6/9	cyt

cp, chloroplast; mt, mitochondrion; cyt, cytosol; n, nucleus.

a for At3g24550 and At5g38560 also a plasmamembrane location was found during large-scale proteomic studies [59-61].

**Table 4:** Characteristics of Novel cpPKs and cpPPs

Accession Number	MW^a^	IP^a^	Domain in Addition to Kinase/Phosphatase Domain	Expression b
cpPKs				
At1g51170	45.7	8.8221	-	At least 3-fold upregulated in response to auxin
At2g34180 (CIPK13, SnRK3.7)	56.7	8.294	NAF domain (necessary and sufficient to mediate interaction with calcineurin B-like calcium sensor proteins)	expressed in roots
**cpPPs**				
At1g07160	40.7	7.3753	-	At least 3-fold upregulated in response to auxin and H_2_O_2_
At2g30020	42.4	7.9156	-	More than 2-fold upregulated in response to wounding and *P. syringae* treatment
At4g33500	78.9	4.375	-	1.5-fold upregulated in response to heat and light + low CO_2_; 1.5-fold downregulated in response to cold
At1g67820	49.4	8.3146	-	2-fold upregulated in response to low nitrate
At2g30170	32.3	4.9442	-	1.5-fold upregulated in response to hypoxia and zeatin
At3g10940	32.1	8.6247	-	1.5-fold upregulated in response to heat and light + low CO_2_; 2-fold downregulated in response to cold
At4g03415	50.3	6.1037	-	Not listed in GENEVESTIGATOR

a calculated molecular weight (MW) in kDa and isolelectric point (IP) according to TAIR database (v7),

b according to GENEVESTIGATOR database [62].
